# Global paediatric surgery: meeting an unmet need—the response of the British Association of Paediatric Surgeons

**DOI:** 10.1007/s00383-018-4365-7

**Published:** 2018-10-20

**Authors:** Kokila Lakhoo, George G. Youngson

**Affiliations:** 1 Oxford University and Oxford University Hospitals, Oxford, UK; 2 Paediatric Surgery, University of Aberdeen, Aberdeen, UK

**Keywords:** Paediatric global surgery, Training, Leadership

## Abstract

**Aim:**

Outline the response from an organisation regarding the unmet needs in global children’s surgery

**Method:**

The burden of global surgical disease, whilst daunting, is becoming increasingly better defined as agencies, surgical colleges and professional specialty associations all attempt to increase capacity in terms of manpower, support education and find sustainable solutions to the deficit of health in treating women and children. However, definition of the problem does not in itself create change and similarly, humanitarian activities including volunteering by established surgical practitioners and other non-governmental organisations (NGOs) make only marginal improvement in the standards of care on offer at a global level.

**Results:**

The International Affairs Committee, British Association of Paediatric Surgeons (BAPS) has had its target firmly set on investing in potential leaders within paediatric surgery in low- and middle-income countries (LMICs), and sharing elements of the educational programme made available for training within the UK and Ireland with the aim of contributing to the solutions of inequity in the surgical standards available to the world’s children.

**Conclusion:**

This article outlines some of the practical steps that have been deployed by BAPS by way of sharing the responsibility for problem-solving at a global level. It also highlights the need for clarity in advocacy and the route through which effective communication can translate into wider and more effective delivery of surgical care for children.

## Introduction

A central tenet to which the British Association of Paediatric Surgeons [1] (BAPS) strives to adhere is the oft-quoted phrase attributable to Sir Denis Browne, the founding father of BAPS, and inscribed on his eponymous medal, viz. “The Aim of Paediatric Surgery Is to Set a Standard Not to Seek a Monopoly”. Nowhere is this maxim more challenged than when applied to the surgical care of children at an international level. Global estimates of the world’s population who do not have access to safe surgery is close to 5 billion people and given that the childhood population (up to 15 years) constitutes almost 50% of low- and middle-income countries, then up 2.5 billion children in those locations do not enjoy the standard of care that is available in the high-income countries.

The scale of this problem, whilst daunting, is becoming increasingly better defined as agencies such as the Lancet Commission [2], the Global Initiative in Children Surgery [3] (GICS), surgical colleges and professional specialty associations all attempt to increase capacity in terms of manpower, support education and find sustainable solutions to the deficit of surgeons, anaesthetists, obstetricians, nurses and allied health professionals treating women and children. However, definition of the problem does not in itself create change and similarly, humanitarian activities including volunteering by established surgical practitioners and other non-governmental organisations (NGOs) make only marginal improvement in the standards of care on offer at a global level. Progress in the four problem areas identified by the Lancet Commission—“stuff, staff, space and systems”—is gradually being made and BAPS has had its target firmly set on investing in potential leaders within paediatric surgery in low- and middle-income countries (LMICs), and sharing elements of the educational programme made available for training within the UK and Ireland with the aim of contributing to the solutions of inequity in the surgical standards available to the world’s children.

This article outlines some of the practical steps that have been deployed by BAPS by way of sharing the responsibility for problem-solving at a global level. It also highlights the need for clarity in advocacy and the route through which effective communication can translate into wider and more effective delivery of surgical care for children.

## International Affairs Committee

The International Affairs Committee was launched in 2002 by Professor D Lloyd during his presidency of BAPS with the aim of “promoting and facilitating the international exchange of professional values in paediatric surgery”. This initiative gave rise to a structure and governance process which allowed BAPS to provide support and accountability and manage the requisite functions of a dedicated committee. The International Affairs Committee (IAC) was, therefore, established, chaired in its early years by Professor George Youngson (2005–2010) and latterly by Professor Kokila Lakhoo (2011—onwards).

The IAC established four main functions.

- Implementation of the International forum—a scientific session focusing on global surgery and held at each Annual International Congress of BAPS.
- Creation of visiting fellowships.
- Creation of sponsored scholarships.
- Provision of neonatal skills courses in LMICs.

## International forum

This meeting is part of the International Annual Congress of BAPS and invites speakers from around the world who have had experience in teaching, training and research in low- and middle-income countries (LMIC). The speakers are mainly senior surgeons but more recently, the forum has extended into presentations by visiting fellows and scholarships from LMIC who share their experiences. In the last 4 years, an eponymous Greenwood lecture has also been delivered as part of the BAPS conference by invited international speakers including Dr. John Sekabira (Uganda, 2014) and Dr. Dan Poenaru (Montréal, Canada, 2015), Dr. Nobhojit Roy (India, 2016), and Dr. Emmanual Makasa, (Zambia, 2017).

## Visiting fellows

Two visiting fellowships have been formulated [4].

*The Greenwood fellowship* Philanthropic financial support from Mr. Hugh Greenwood OBE, a generous benefactor of BAPS, has allowed selected surgeons from LMICs to visit a range of UK paediatric surgical institutions for a period not extending beyond 3 months and attend the BAPS conference. The aims of this attachment are not only to observe contemporary clinical practice as delivered by the UK and Irish paediatric surgeons but also to observe current methods and trends in surgical education and identify what might be relevant in the fellows’ own country. These Greenwood fellows are listed in Table 1.

**Table 1:** Greenwood fellows 2005–2017

Year	Greenwood fellow	Visitor country	UK centre hosting Greenwood fellow
2018	Dr. Feseha Temesgen	Ethiopia	Liverpool paediatric urology
2017	Dr. Ritesh Shrestha	Nepal	St. Georges Hospital oncology
2016	Dr. Taurai Zimunhu	Zimbabwe	Visa refused: award awaiting
2015	Dr. Erika Gacus	Philippines	Oxford and Evelina: tumour board; laparoscopy
2014	Dr. Mohajeran	Iran	Evelina: Neonatal surgery
2013	Dr. Rupam Talukder	Bangladesh	Norwich: laparoscopy and neonatal surgery
2012	Dr. Adesoji O Ademuyiwa	Nigeria	King’s: neonatal and laparoscopic surgery
2011	Dr. Mohammed Iqbal	Pakistan	London: laparoscopy
2010	Dr. Philemon Okoro	Nigeria	GOS and Norwich: urology and laparoscopy
2009	Dr. Ramnandan Chaudhary	Nepal	Norwich: general paediatric surgery
2008	Dr. Abdulla Farooq	Bangladesh	Norwich: paediatric surgery skills
2007	Dr. Emmanual Ameh	Nigeria	King’s & GOS: administrative skills
2006	Dr. Sam Mhando	Tanzania	Oxford: leadership and administration
2005	Dr. John Sekabira	Uganda	Durban South Africa: paediatric surgery skills

*The Lister fellowship* A legacy from Professor J Lister has funded a number of visiting surgeons to attend the international forum. This subsidy deals with all registration travel and accommodation requirements.

In both cases, nominations for these fellowships are made from the appropriate agencies (either colleges or specialty associations) in the regions involved and nominations come from Africa, Asia, Southeast Asia, and the Middle East with attempts made by IAC to alternate placement on a rotational/regional basis. The Africa awards are pre-selected by Pan African Paediatric Surgery Association (PAPSA), the Asia region by the SAARC Association which includes India, Pakistan, Nepal, Bangladesh and Sri Lanka, the Southeast Asia region by ASEAN Society of Paediatric Surgeons which includes countries, namely Thailand, Laos, Cambodia, Myanmar, Malaysia, Singapore, and Philippines and the Middle East Association of Paediatric Surgeons which includes Iran, Iraq, Afghanistan, Kuwait, Oman and Saudi Arabia.

A consistent and as-yet unresolved problem, however, has been the inability of delegates to secure visiting visas into the United Kingdom in spite of strong advocacy and interventions from both BAPS executive and IAC to contend with this matter.

## Visiting scholars

Since 2009, up to three scholars per year have been invited from the above three jurisdictions to attend the BAPS international conference without registration or accommodation costs. This is partially funded by BAPS and by the Greenwood donation. A precondition of selection for a scholarship is delivery of the short paper at the international forum and/or presentation of the poster (Table 1).

## International skills courses

Simulation skills courses have been run in neonatal surgery in a number of venues (see Table 2). Implicit in these courses is an attempt to teach aspiring paediatric and general surgeons both the knowledge behind and the practical skills involved in treating neonatal surgical conditions. Oesophageal atresia repair, duodenal atresia repair, pyeloplasty, insertion of gastrostomies, suprapubic catheters and chest tube, as well as end to back anastomosis for diameter discrepancy, are examples of the type of procedures carried out by local delegates using locally acquired materials and led by Mr. Tony Lander, Birmingham.

**Table 2:** Hugh Greenwood skills course 2010–2018

Year	Country	Organisation link	Course	Number of attendees
2018	Ethiopia	PAPSA	Neonatal skills course	35
2017	Iran	MEPSA	Laparoscopic skills course	45
2017	Sudan	Sudanese association	Advance neonatal skills course	25
2017	Mozambique	COSECSA	Paediatric skills course	30
2017	Philippines	ASEAN	Neonatal skills course	27
2017	Pakistan	Asia meeting	Laparoscopic skills course	32
2016	Nigeria	PAPSA	Neonatal skills course	42
2016	Pakistan	Asia meeting	Laparoscopic skills course	16
2016	Nigeria	PAPSA	Laparoscopic skills course	42
2015	Malawi	COSECSA (clinical officers)	Paediatric skills course	18
2015	Pakistan	Asia meeting	Laparoscopic skills course	35
2014	Egypt	PAPSA	Neonatal skills course	72
2012	South Africa	PAPSA	Neonatal skills course	28
2012	Ghana	PAPSA	Neonatal skills course	25
2012	Ethiopia	COSECSA	Neonatal skills course	27
2010	Tanzania	PAPSA	Neonatal skills course	32
2010	Tanzania	PAPSA	Laparoscopic skills course	15

These courses have again been funded through the generosity of Mr. Hugh Greenwood and his family (Table 2).

## Other functions

Advocacy remains an important additional element of the work of the IAC and it has encouraged trainees to utilise “out of programme” experiences in LMICs and has also taken part in the organisation of those attachments (BAPS traveller). So far two awards have been granted with one candidate having spent a year in Johannesburg, South Africa, as a supernumerary trainee and the other ran a gastroschisis training project in Ivory Coast, Uganda, South Africa, Malawi and Nigeria. Both these trainees have now taken interest in Global surgery as part of their future career.

IAC members support and help administer Global Initiative in Children Surgery (GICs) which is a consortium of 94 countries attempting to establish standards of care, resources and a research framework that will address the challenges of global surgery across the globe and with countries with variable contexts in terms of facilities, fiscal considerations and available manpower.

## Discussion

Many of the collaborations in global health have been focused on collaborations between high-income and low-income countries. Collaborations between low-income countries have not received as much attention or support, partly because most resources or programs come from high-income countries. Following the Lancet meeting, GICS was established to bring together surgical staff from low-income countries to come together and establish universal standards of facilities and resources required of health care institutions internationally, to provide surgical care for children at a variety of levels of dependence and acuity of their surgical condition [5]. However, another important request from the LMIC region was short-term training in surgical specialities in a better established region in LMIC (“South to South Training”). Emphasis was on regular short-term training to allow for short-term absence from the home country and avoid loss of trainees to better resourced areas within LMIC. The first South to South Training candidate was Dr. John Sekabira from Uganda who was supported by The Hugh Greenwood Children’s Research Fund to train in paediatric Surgery in Durban, South Africa. Subsequently from funds raised by individual members of the IAC, we have further supported candidates from Malawi, Tanzania, Ghana, and Ivory Coast to receive training in South African paediatric surgical centres.

The IAC continues to liaise with other organisations so that its work is not duplicated but complementary to other organisations. Liaisons have been formed with all the surgical Royal Colleges within the United Kingdom and Ireland, European Paediatric Surgical Association (EUPSA), Canadian Association of Paediatric Surgeons (CAPS), American Paediatric Surgical Association (APSA), Australasian Association of Paediatric Surgeons (AAPS), Global Initiative for Children’s Surgery (GICS) and more closely with the regions through which our courses, fellowships and scholarships are linked, namely PAPSA, COSECSA, ASEAN group, SAARC group and the Middle Eastern Association.

Trainee involvement in international affairs committee has been rigorous. A trainee representative of the IAC engages our BAPS trainees with those from some HIC but mainly from LMIC. These associations have resulted in trainee exchange with LMIC, joint research projects and joint training projects. The trainee representative is the key contact point for our international scholars and fellows.

Measuring the impact of these initiatives is difficult on a number of counts. The scale and complexity of the factors contributing to the deficit in paediatric surgical care in LMIC is so vast that any one action—however beneficial—will only produce marginal change in shifting the burden of surgical care in children, albeit in the right direction. Studies itemising the return on investment in provision of facilities have shown impressive efficiency [6] but statistical analysis is redundant in this field of medicine because of a lack of contextual reference points and proxies need to suffice instead. Surrogates such as retention and growth of the workforce and initiatives which attempt to register the characteristics and volume of surgical disease [7] in LMIC will bring some definition against which change and impact can be measured in the future. In the meantime, and in the absence of any directly linear causal relationship between the actions of bodies such as BAPS and our sister organisations, we should commit to meeting the challenges of supporting paediatric surgical care globally, through advocacy and action, and meet the aspiration of the Denis Browne maxim.

## Conclusion

The International Affair Committee of BAPS through its global activities and collaborations have aimed to develop leaders, share skills, share educational programs and support trainees both from BAPS and LMIC to collaborate and attain skills towards setting standards and improving the care of the global surgical child.
